# Effects of experimental warming on stomatal traits in leaves of maize (*Zea may* L.)

**DOI:** 10.1002/ece3.674

**Published:** 2013-08-01

**Authors:** Yunpu Zheng, Ming Xu, Ruixing Hou, Ruichang Shen, Shuai Qiu, Zhu Ouyang

**Affiliations:** 1 Key Laboratory of Ecosystem Network Observation and Modeling, Institute of Geographic Sciences and Natural Resources Research, Chinese Academy of Sciences11A Datun Road, Beijing, 100101, China; 2 University of Chinese Academy of SciencesBeijing, 100039, China; 3 Center for Remote Sensing and Spatial Analysis, Department of Ecology, Evolution and Natural Resources, Rutgers University14 College Farm Road, New Brunswick, New Jersey, 08901; 4 Yucheng Comprehensive Experimental Station, Chinese Academy of SciencesBeijing, 100101, China

**Keywords:** Elevated temperature, maize (*Zea may* L.), Ripley's *K*-function, spatial distribution pattern, stomatal aperture size and shape, stomatal frequency

## Abstract

We examined the warming effects on the stomatal frequency, stomatal aperture size and shape, and their spatial distribution pattern of maize (*Zea may* L.) leaves using a light microscope, an electron scanning microscope, and geostatistic techniques. A field manipulative experiment was conducted to elevate canopy temperature by 2.08°C, on average. We found that experimental warming had little effect on stomatal density, but significantly increased stomatal index due to the reduction in the number of epidermal cells under the warming treatment. Warming also significantly decreased stomatal aperture length and increased stomatal aperture width. As a result, warming significantly increased the average stomatal aperture area and stomatal aperture circumference. In addition, warming dramatically changed the stomatal spatial distribution pattern with a substantial increase in the average nearest neighbor distance between stomata on both adaxial and abaxial surfaces. The spatial distribution pattern of stomata was scale dependent with regular patterns at small scales and random patterns at larger scales on both leaf surfaces. Warming caused the stomatal distribution to become more regular on both leaf surfaces with smaller *L*(*t*) values (Ripley's *K*-function, *L*(*t*) is an expectation of zero for any value of *t*) in the warming plots than the control plots.

## Introduction

Stomata are the pores on a leaf surface controlling gas exchanges, mainly CO_2_ and water vapor, between the atmosphere and plants (Woodward 1987; Hetherington and Woodward 2003), and thus regulate carbon and water cycles in various ecosystems (Franks and Beerling 2009; Haworth et al. 2010; Taylor et al. 2012). Globally, the gas exchanges between leaf surface and the atmosphere are massive at *c*. 440 × 10^15^ g CO_2_ per year through photosynthesis and 32 × 10^18^ g H_2_O per year through leaf transpiration (Ciais et al. 1997; Hetherington and Woodward 2003; Lake and Woodward 2008). Plant leaves usually optimize their gas exchange by altering stomatal pore openness, stomatal aperture size, stomatal frequency (stomatal density and stomatal index), and stomatal distribution pattern, which are regulated by both environmental factors (Lake et al. 2002; Hetherington and Woodward 2003; Schlüter et al. 2003; Casson and Gray 2008; Lake and Woodward 2008; Franks and Beerling 2009) and genetic signals (Bergmann 2004; Liang et al. 2005; Shpak et al. 2005; Hara et al. 2007; Lampard et al. 2008; Hunt and Gray 2009; Hunt et al. 2010; Kondo et al. 2010; Sugano et al. 2010). In connection with the studies of photosynthetic acclimation under climate warming, stomatal response is of special interest, because stomatal traits set the limit for maximum stomatal conductance for gas exchange and thus has a potential to affect carbon gain and water use efficiency (Beerling 1997; Luomala et al. 2005).

Leaf maximum stomatal conductance has been widely used to quantify gas exchange efficiency, which is dependent on stomatal aperture size and shape, frequency, and distribution pattern (Buckley et al. 1997; Hetherington and Woodward 2003; Franks and Beerling 2009; Franks et al. 2009). Usually plants respond quickly to short-term environmental changes by changing the openness of the stomatal pore, a response also known as stomatal movement (Sharkey and Raschke 1981; Kwak et al. 2001; Guo et al. 2003; Young et al. 2006; Shimazaki et al. 2007; Shang et al. 2009). Many studies have shown that stomatal movement is controlled by light (Humble and Hsiao 1970; Sharkey and Raschke 1981; Kwak et al. 2001; Takemiya et al. 2006), CO_2_ concentration (Ogawa 1979; Young et al. 2006; Lammertsma et al. 2011), temperature (Honour et al. 1995; Feller 2006; Reynolds-Henne et al. 2010), drought stress (Guo et al. 2003; Klein et al. 2004), air humidity (Lange et al. 1971; Schulze et al. 1974), and ultraviolet light (Herčík 1964; Eisinger et al. 2000). In addition to responses to short-term environmental changes through stomatal movement, long-term (decadal) environmental changes such as climate warming may also affect individual stomatal aperture size, stomatal frequency, and stomatal distribution pattern (Anderson and Brisk 1990; Lammertsma et al. 2011).

So far, no consistent conclusions have been drawn on the effect of warming on stomatal traits in the literature. Most studies found that warming had little effect on stomatal density and stomatal index (Apple et al. 2000; Hovenden 2001; Kouwenberg et al. 2007; Fraser et al. 2009), while other studies found that warming could decrease stomatal density (Beerling and Chaloner 1993) and index (Ferris et al. 1996) or increase stomatal density (Reddy et al. 1998; Xu et al. 2009) and stomatal index (Xu and Zhou 2005). In addition, warming could also change individual stomatal aperture size and shape (Ferris et al. 1996; Zuo et al. 2005; Zhang et al. 2010). For example, Ferris et al. (1996) found that experimental warming substantially increased the stomatal aperture length of a perennial ryegrass (*Lolium perenne*). By contrast, a more recent study reported that warming significantly decreased stomatal aperture length of four alpine meadow species including *Thalictrum alpinum, Kobresia humilis*, *Gentiana straminea*, *Elymus nutans* in the Qinghai-Tibetan plateau, China (Zhang et al. 2010).

In addition to the number, size and shape of stomata on the leaf surface, warming may also alter the spatial distribution pattern of stomata through cell division and cell differentiation (Croxdale 1998, 2000; Berger and Altmann 2000; Shpak et al. 2005) which are regulated by genetic signals (Nadeau and Sack 2002; Bergermann et al. 2004; Juarez et al. 2004; Shpak et al. 2005; Wang et al. 2007; Hunt et al. 2010) and environmental factors (Wang et al. 2007; Casson and Gray 2008) during stomatal development stages. The pattern of stomatal distribution is highly variable among species and recent advances in genetic studies have found that a number of genes, such as *SDD1*, *EPF1,* the putative receptors *TMM,* and the *ERECTA*-gene family, are involved in the determination of stomatal spacing (Nadeau and Sack 2002; Hunt et al. 2010). The spatial variation of stomata can be characterized at multiple scales, such as the adaxial versus abaxial surface, variations among different leaf sections, and the association/aggregation of individual stomata on a single leaf surface. Earlier studies have reported that stomatal density significantly differed between the adaxial and abaxial surfaces (Ciha and Brun 1975; Green et al. 1990; Ferris et al. 1996, 2002; Croxdale 1998, 2000; Reddy et al. 1998). Meanwhile, the distribution of stomata between leaf surfaces is associated with acclimation and adaptation to environmental factors such as temperature, water stress, light exposure, and CO_2_ concentration (Parkhurst 1978; Mott et al. 1982; Ceulemans et al. 1995; Smith et al. 1998; Ferris et al. 2002; Driscoll et al. 2006; Soares et al. 2008). Moreover, the changes in the adaxial/abaxial ratio of stomata may also alter leaf function such as photosynthesis, because the stomata in the adaxial and abaxial leaf surfaces feature specific responses to environmental stresses such as CO_2_ and temperature, thus result in the changes in leaf photosynthesis. Previous studies have found that growth at high CO_2_ altered the regulation of photosynthesis on the adaxial and abaxial leaf surfaces of maize (*Zea mays*) (Driscoll et al. 2006) and *Paspalum dilatatum* (Soares et al. 2008) due to the changes in adaxial/abaxial ratio of stomata between leaf surfaces. In addition, the spatial variation of stomatal distribution was also seen among different leaf sections, such as the leaf tip, middle, and base section (Salisbury 1927; Sharma and Dunn 1969; Tichá 1982; Smith et al. 1989; Ferris et al. 1996; Zacchini et al. 1997; Stancato et al. 1999; Xu et al. 2009). However, several previous studies investigated stomatal features only collecting samples at the middle section of the abaxial or abaxial leaf surface (Beerling and Chaloner 1993; Hovenden 2001; Xu and Zhou 2005; Kouwenberg et al. 2007).

There are three photosynthetic pathways in terrestrial plants including C_3_, C_4_, and crassulacean acid metabolism (CAM). Globally, most plant species use the C_3_ photosynthetic pathway, which is characterized by a low photosynthetic efficiency, because the process is compromised by photorespiration (Osborne and Freckleton 2009). However, C_4_ pathway represents evolutionary advancements over the ancestral C_3_ pathway (Ehleringer et al. 1997) due to high rates of photosynthesis and efficient use of water and nitrogen (Wang et al. 2009). It is noted that the performance of each pathway is significantly influenced by environmental conditions such as temperature (Ehleringer et al. 1997). Given the morphological and biochemical innovation, C_4_ plants are proposed to better adapt to warming conditions than their C_3_ counterparts (Dwyer et al. 2007; Sage and Kubien 2007). Maize (*Zea mays* L.) is an economically important food crop, which also uses C_4_ photosynthetic pathway. So far, most studies mainly focused on the responses of leaf photosynthesis of maize plants to nitrogen (Muchow and Sinclair 1994; Correia et al. 2005), drought stress (Dwyer et al. 1992; Earl and Davis 2003), CO_2_ concentration (Driscoll et al. 2006; Leakey et al. 2006), and salt stress (Khodary 2004; Sheng et al. 2008). To our knowledge, however, few studies have been reported investigating warming effects on the adaxial/abaxial ratio, the variation of stomata on different leaf sections, and the stomatal distribution pattern on single leaf surfaces of maize plants. The objectives of the current study are to examine warming effects on: (1) stomatal frequency; (2) stomatal aperture size; and (3) stomatal distribution pattern in maize leaves through a field warming experiment in northern China.

## Materials and Methods

### Site description

This study was conducted in the Yucheng Comprehensive Experiment Station (36°40′–37°12′ N, 116°22′–116°45′ E; an elevation of 28 m) which is located in the lower reach of the Yellow River in the North China Plain. The study area features a typical monsoon climate with average annual precipitation of 610 mm and annual mean temperature of 13.1°C. Approximately 70% of the annual precipitation is received between June and September (Hou et al. 2012). The soil at the station is classified as Calcaric fluvisols in the FAO-Uneson system with 66% silt; 22% clay; and 12% sand. The soils are chemically characterized with an average pH value of 8.5, organic matter content of 1.47 g/kg, and total N, P, and K concentration of 0.9 g/kg, 0.2% and 2.26%, respectively. A double cropping system with winter wheat (*Triticum aestivum* L.) and summer maize (*Zea mays* L.) has been practiced in this area for at least 50 years (Zhang and Ren 2012).

### Warming experiment

The warming experiment, initiated in September 2009, consists of six 3 × 4 m plots with three of the plots as warming plots and the other three plots as control (three replicates). A 5 m buffer was established between the plots to reduce disturbances. The warming plots have been heated continuously since November 18th, 2009 using infrared radiators with a dimension of 165 × 1.5 cm in length and width (Kalglo Electronics Inc, Bethlehem, PA). Each infrared heater was suspended 2.25 m above the ground. A reflector associated with the heater can be adjusted so as to generate an evenly distributed radiant input to the plant canopy (Kimball 2005). In the control plot, one “dummy” heater with the same shape and size as the infrared radiator was suspended at the same height to eliminate shading effects of the infrared radiator.

Air temperature at 2.4 m above the ground and soil temperature at 5 cm depth were continuously monitored with thermocouple sensors and the averages were recorded hourly with PT 100 thermocouples (Unism Technologies Incorporated, Beijing, China). The foliar surface temperature was measured using a portable infrared thermometer (FLUKE 574; Fluke Inc., Carlsbad, CA). The warming, on average, has increased air, soil, and canopy temperature by 1.42 ± 0.18/1.77 ± 0.24 (day/night), 1.68 ± 0.9/2.04 ± 0.16 (day/night), 2.08 ± 0.72 (day), respectively, in comparison with the control during the maize growth period from June 24th to October 7th of 2011. Soil moisture (0–10 cm average), was also monitored in the middle of each plot with a FDS100 soil moisture sensor (Unism Technologies Incorporated, Beijing, China). All the plots were irrigated with normal management schedules to ensure that the soil moisture was not a limiting factor to plant growth. During the maize growth period the soil moisture (% by volume) in the top 10 cm averaged 26.02 ± 0.86% in the control plots and 25.04 ± 0.52% in the warming plots. Moreover, no significant difference was detected in the relative air humidity between the ambient and warming plots (data not shown).

In order to promote uniform germination, the seeds (cv. Zhengdan 958) of maize (*Zea may* L.) were treated at 4°C in dark and wet environments for 2 days before they were sowed. Afterwards, the seeds were sown in the control and warming plots on June 24th, 2011. Given that the ear leaf of each plant is the most important for determining the maize yield, five fully expanded ear leaves were randomly collected, namely five plants in each treatment for field measuring and sampling on August 24th, 2011, 60 days after sowing.

### Field gas exchange measurements and sampling

We randomly selected five maize plants grown in the three control plots or the three warmed plots for gas exchange measurements. Specifically, we selected maize plants from plot 1 (two plants), plot 2 (two plants), and plot 3 (one plant) among the three ambient plots. We also selected five maize plants from the three warmed plots with the same number of maize plants as the ambient plots; namely, two plants (plot 1), two plants (plot 2), and one plant (plot 3) were selected among the three warmed pots, respectively. Each fully expanded ear leaf of the five selected plants was used for the measurements of stomatal conductance (Gs) and transpiration rates (Tr) using a portable photosynthesis system (LI-6400; LI-COR Inc., Lincoln, NE). The measurements were conducted with leaf temperature at 30°C, PAR at 1500 μmol/m^2^/s, CO_2_ concentration at 380 μmol/mol, and cuvette vapor pressure deficit (VPD) at 2.0 KPa. The data of stomatal conductance (Gs) and transpiration rates (Tr) were tested using one-way analysis of variance (ANOVA) followed by Duncan's multiple range test (*P* < 0.05).

To characterize the maximum stomatal pore size, we collected the stomatal samples at optimal conditions with a temperature of *c*. 30°C at a sunny day (August 24th, 2011) during 10:30–11:00 am. We sampled separately from the tip, middle, and base sections of the adaxial and abaxial surfaces using a colorless nail polish. The adaxial and abaxial epidermis of the leaves were cleaned first by a degreased cotton ball and then carefully smeared with nail varnish from the mid-area between the central vein and the leaf edge for about half an hour. The thin film (approximate 5 by 15 mm) was peeled off from the leaf surface and mounted on a glass slide. Then the thin film was immediately covered with a cover slip and pressured lightly with a fine-point tweezers. We used the same sampling method as in previous studies on the topic (Radoglou and Jarvis 1990; Ferris et al. 1996; Reddy et al.*,* 2001; Xu and Zhou 2005; Xu et al. 2009; Zhang et al. 2010).

### Laboratory measurements

The imprints were observed and photographed in the laboratory with a microscope (DM2500; Leica Corp, Biberach, Germany) equipped with a digital camera (DFC 300-FX; Leica Corp). We took 15 images from five microscopic fields at each section (tip, middle, and base section) on the adaxial and abaxial surfaces of the five leaves sampled from the three replicated control or warmed plots. Then we randomly selected five images (subsamples) from each leaf section of the adaxial or abaxial surface per leaf (five subsamples* three sections* five leaves = 75 samples). Because the three ambient plots or the three warmed plots are the three replicates, all the data of the stomatal traits from the five sampled leaves (75 samples) were averaged within each plot, namely 30 samples (five subsamples* three sections* two leaves = 30 samples) from plot 1 or plot 2 and 15 samples (five subsamples* three sections* one leaf = 15 samples) from plot 3 in the three ambient plots or the three warmed plots. Moreover, we combined the subsamples of the adaxial and abaxial surfaces for estimating the stomatal characteristics for the whole leaf in the control or warmed plot (5 subsamples * 3 sections * 2 surfaces * 5 leaves = 150 samples). Stomata and epidermal cells were counted on the images. Stomatal density (SD), epidermal cell density (ECD), and stomatal index (SI) were calculated according to the methods outlined by Ceulemans et al. (1995) and Teng et al. (2006). Specifically, stomatal density (SD) and ECD were expressed as the number of stomata and epidermal cells per unit leaf area. The stomatal index (SD) was estimated as the percentage of stomata/(epidermal cells + stomata) × 100% (Xu and Zhou 2005; Xu et al. 2009). To characterize the features of length, width, and area of stomatal pores and epidermal cells, we randomly selected six stomata and six epidermal cells from the above selected images (75 subsamples * six stomata/epidermal cells = 450 samples per leaf surface for stomatal pores or epidermal cells) for measuring stomatal apertures length (SAL), stomatal apertures width (SAW), stomatal apertures area (SAA), stomatal apertures circumference (SAC) and epidermal cell length (ECL), epidermal cell width (ECW), epidermal cell area (ECA), and epidermal cell circumference (ECC) with the Image J quantification software (NIH, Bethesda, MD). In addition, we also calculated stomatal aperture area index (SAAI) and stomatal aperture shape index (SASI). The SAAI is defined as the total stomatal aperture area per unit leaf area calculating as stomatal average density × stomatal aperture area per stoma × 100%. The SASI is calculated by the function that shape 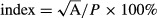, where A is the stomatal aperture area and P is the stomatal aperture circumference. Both the stomatal traits (SD, SI, SAL, SAW, SAA, SAC, SAAI, and SASI) and epidermal features (ECD, ECL, ECW, ECA, and ECC) were statistically analyzed with one-way analysis of variation (ANOVA) followed by Duncan's multiple range test (*P* < 0.05).

To measure the spatial distribution patterns of stomata on both the adaxial and abaxial surfaces, we randomly sampled two pieces (2 × 2 mm) in the middle section of each ear leaf from three maize plants grown under ambient or elevated temperature. These samples were treated with a fixative solution consisting of 2.5% (v/v) glutaraldehyde (in 0.1 m phosphate buffer, pH 7.0). Samples were stored at 4°C and transported to the laboratory immediately. Then, the samples were washed six times with the same buffer and postfixed in 1% (v/v) osmium tetroxide for 3 h at room temperature. After being washed with the same buffer, leaf tissues were passed through an ethanol dehydration series. Then the samples were critical point-dried, mounted on stubs, and coated with gold in a high-vacuum evaporation unit. The samples were examined and photographed at 10 KV under a Quanta 200 scanning electron microscope (FEI Corp, Hillsboro, OR).

### Spatial pattern analysis

We randomly selected three scanning electron micrographs (a magnification of 100) from three maize leaves on each leaf surface to determine the spatial distribution pattern of stomata. For this analysis, we treated each stoma as a single point on the leaf surface by using the center of each aperture as the focal point. The selected micrographs were first digitized with a GIS software (ArcGIS 10.0; ESRI Inc., Redlands, CA). Then, the point pattern analysis was conducted with the Ripley's *K*-function, a cumulative density function using the second moment of all point-to-point distances to evaluate two-dimensional distribution patterns at different scales (Ripley 1976). The results were plotted in the *L*(*t*) values as shown below:


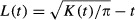
(1)

where *L*(*t*) is an expectation of zero for any value of *t* when the pattern is Poisson random (Skarpe 1991; Haase et al. 1997). In order to estimate the boundaries of the 95% confidence level, we used the Monte Carlo simulation by running a random distribution for 1000 times. If the stomata are randomly distributed on the leaf surface at a given scale of *t*, then the calculated *L*(*t*) value should be located within the 95% boundaries. If the *L*(*t*) value is greater than the upper 95% boundary, then the stomata will follow a cluster distribution at that scale. Otherwise, the stomata would follow a regular distribution at the scale if the *L*(*t*) value is smaller than the lower 95% boundary. Details of the Ripley's *K* analysis can be found in Diggle (1983). The differences of the minimum *L*(*t*) values of both leaf surfaces between the ambient plots and the warmed plots were statistically compared by one-way analysis of variation (ANOVA) followed by Duncan's multiple range test (*P* < 0.05).

### Statistical analysis

The warming effects on the features of stomata (SD, SI, SAL, SAW, SAA, SAC, SAAI, and SASI) and epidermal cells (ECD, ECL, ECW, ECA, and ECC) were tested using one-way analysis of variation (ANOVA) followed by Duncan's multiple range test (*P* < 0.05). To estimate the interaction effects of temperature, leaf surface, and leaf section on the stomatal traits (SD, SI, SAL, SAW, SAA, SAC, SAAI, and SASI), we used three-way ANOVA followed by Duncan's multiple range test (*P* < 0.05). All statistical analyses were performed using the *SPSS* 13.0 software (Chicago, IL).

## Results

### Stomatal frequency

Experimental warming had little effect on stomatal density (SD) on both leaf surfaces and sections except for the base section on the adaxial surface and the tip section on the abaxial surface (Tables 1 and 2). We found that the SD was significantly different between the adaxial and abaxial surfaces with an average stomatal density of 56(5) stomata/mm^2^ on the adaxial surface and 77(3) stomata/mm^2^ on the abaxial surface of maize leaves grown under ambient temperature. While warming barely increased the SD from 56(5) to 58(9) stomata/mm^2^ on the adaxial surface and 77(3) to 81(1) stomata/mm^2^ on the abaxial surface. Moreover, we found that experimental warming significantly increased the SD by 19.6% (*P* = 0.023, *F* = 8.363, df = 149) from 56(3) stomata/mm^2^ to 67(9) stomata/mm^2^ at the base section on the adaxial surface and by 11.0% (*P* < 0.001, *F* = 14.901, df = 149) from 73(1) stomata/mm^2^ to 81(1) stomata/mm^2^ at the tip section on the abaxial surface, while no significant difference was detected at other sections on the adaxial or abaxial surfaces (all *P* > 0.05). Our results also showed that the warming effect on the stomatal index (SI) was divergent on the adaxial and abaxial surfaces, where warming significantly increased the SI by 19.6% on the adaxial surface (*P* < 0.001, *F* = 10.024, df = 299) but only 6.2% on the abaxial surface (*P* = 0.098, *F* = 6.954, df = 299; Table 1). Similarly, experimental warming also had different effect on the SI among various leaf sections (Table 2). We found that warming significantly increased the SI by 23.6% at the middle section (*P* = 0.014, *F* = 8.829, df = 149) and by 37.9% at the base section (*P* = 0.003, *F* = 14.921, df = 149) of the adaxial surface and by 10.2% (*P* = 0.025, *F* = 3.220, df = 149) at the middle section of the abaxial surface (Table 2). However, experimental warming had little effect on the SI at the tip section on the adaxial surface and at the tip and base sections on the abaxial surface (all *P* > 0.05; Table 2).

**Table 1:** Effects of experimental warming on stomatal features of maize leaves

Parameters	Ambient temperature		Elevated temperature		Increase (%)	*P*-value
	
	Adaxial	Abaxial	Adaxial	Abaxial		
SD (stomata/mm^2^)	56 (5)^b^	77 (3)^a^	58 (9)^b^	81 (1)^a^	–	–
	67		70		4.5	*P* = 0.826
SI (%)	13.8 (0.1)^c^	19.4 (0.5)^ab^	16.5 (0.3)^b^	20.6 (0.8)^a^	–	–
	16.6		18.6		11.7	*P* = 0.012
SAL (μm)*	36.2 (2.9)^a^	35.5 (1.5)^a^	31.0 (2.7)^b^	28.5 (3.0)^b^	–	–
	35.9		29.8		−17.0	*P* < 0.001
SAW (μm)*	3.6 (0.4)^bc^	3.2 (0.7)^c^	4.6 (0.7)^ab^	4.5 (0.9)^a^	–	–
	3.4		4.6		33.8	*P* < 0.001
SAA (μm^2^)	119 (10)^bc^	100 (19)^c^	150 (25)^a^	135 (19)^ab^	–	–
	110		143		30.1	*P* < 0.001
SAC (μm)	75 (9)^bc^	70 (4)^c^	87 (14)^a^	77 (7)^b^	–	–
	73		82		13.1	*P* = 0.006
SAAI (%)	0.66 (0.24)^c^	0.77 (0.12)^bc^	0.87 (0.26)^b^	1.10 (0.25)^a^	–	–
	0.71		0.99		39.9	*P* < 0.001
SASI (%)	14.7 (2.1)^a^	14.3 (1.7)^a^	14.5 (2.4)^a^	15.1 (2.2)^a^	–	–
	14.5		14.8		2.1	*P* = 0.81

Values given are means ± standard deviation for SD, SI, SAAI, and SASI (75 subsamples and three replicates), and for SAL, SAW, SAA, and SAC (450 subsamples and three replicates). Mean values were compared by the one-way analysis of variance (ANOVA) at *P* < 0.05. Different letters indicate *P* < 0.05 and the same letters indicate *P* > 0.05. SD, stomatal density; SI, stomatal index; SAL, stomatal aperture length; SAW, stomatal aperture width; SAA, stomatal aperture area; SAC, stomatal aperture circumference; SAAI, stomatal aperture area index; SASI, stomatal aperture shape index. *Stomatal aperture length is the longest dimension, and the stomatal aperture width is the widest dimension.

**Table 2:** Warming effects on stomatal and epidermal cell characteristics at different leaf sections of maize

Features	Adaxial surface						Abaxial surface					
	
	Ambient temperature			*Elevated temperature*			Ambient temperature			Elevated temperature		
			
	Tip	Middle	Base	Tip	Middle	Base	Tip	Middle	Base	Tip	Middle	Base
SD	51 (5)^c^	61 (3)^b^	56 (3)^b^	50 (2)^c^	58 (4)^b^	67 (9)^a^	73 (1)^b^	79 (7)^ab^	79 (5)^ab^	81 (1)^a^	82 (12)^a^	80 (2)^ab^
ECD	330 (27)^c^	375 (30)^b^	350 (35)^bc^	329 (40)^c^	277 (21)^a^	283 (18)^a^	309 (28)^ab^	324 (19)^a^	330 (28)^a^	331 (16)^a^	298 (20)^b^	309 (11)^ab^
SI	13.4 (1.8)^b^	14.0 (1.6)^b^	14.0 (2.2)^b^	13.2 (1.9)^b^	17.3 (2.3)^a^	19.3 (2.5)^a^	19.2 (1.3)^b^	19.7 (2.2)^b^	19.4 (1.0)^b^	19.7 (0.4)^b^	21.7 (1.6)^a^	20.6 (0.9)^ab^
SAL	35.6 (2.3)^ab^	35.1 (1.9)^ab^	38.1 (3.8)^a^	30.0 (1.6)^c^	29.5 (2.2)^c^	34.4 (1.5)^b^	36.5 (0.9)^a^	34.8 (1.1)^a^	35.2 (2.0)^a^	28.7 (3.3)^b^	29.6 (2.9)^b^	27.1 (2.7)^b^
SAW	3.4 (0.2)^b^	3.7 (0.4)^b^	3.7 (0.3)^bc^	4.2 (0.3)^ab^	4.9 (1.0)^a^	4.5 (0.3)^ac^	2.9 (0.6)^c^	3.1 (0.6)^c^	3.5 (0.7)^b^	3.9 (0.4)^bc^	4.6 (1.1)^ab^	5.3 (0.4)^a^
SAA	112 (4)^b^	117 (10)^b^	126 (9)^b^	121 (12)^b^	157 (20)^a^	168 (13)^a^	84 (9)^d^	97 (15)^d^	120 (11)^c^	120 (9)^bc^	136 (10)^b^	156 (17)^a^
SAC	74 (7)^a^	77 (13)^a^	73 (7)^a^	95 (9)^b^	81 (9)^a^	83 (11)^ab^	71 (4)^bc^	70 (4)^bc^	69 (3)^b^	74 (5)^bc^	84 (8)^a^	76 (7)^c^
SAAI	0.57 (0.02)^d^	0.71 (0.06)^c^	0.71 (0.05)^c^	0.60 (0.06)^d^	0.91 (0.11)^b^	1.14 (0.09)^a^	0.61 (0.06)^e^	0.77 (0.12)^d^	0.95 (0.09)^c^	0.98 (0.07)^c^	1.11 (0.08)^b^	1.25 (0.14)^a^
SASI	14.4 (1.7)^ab^	14.3 (2.4)^b^	15.5 (2.1)^b^	11.7 (1.4)^a^	15.6 (2.0)^b^	15.7 (1.4)^b^	12.9 (1.0)^c^	14.1 (1.7)^bc^	15.9 (1.2)^a^	15.0 (1.3)^ab^	13.9 (1.2)^b^	16.5 (2.2)^a^

Values given are means ± standard deviation for SD, ECD, SI (25 subsamples and three replicates) and for SAL, SAW, and SAA (150 subsamples and three replicates). Mean values were compared by the ANOVA followed by Duncan's multiple range test, and the different letters represent statistical differences at *P* < 0.05. SD: Stomatal density (number per mm^2^); ECD: Epidermal cell density (number per mm^2^); SI: Stomatal index (%); SAL: Stomatal aperture length (μm); SAW: Stomatal aperture width (μm); SAA: Stomatal aperture area (μm^2^); SAAI: Stomatal aperture area index (%).

In addition to the stomatal frequency of leaf surfaces and sections, experimental warming also had different effects on the adaxial/abaxial ratio of stomatal density and stomatal index (Table 3). We found that warming significantly increased SI from 0.71 to 0.80 (*P* = 0.028, *F* = 4.738, df = 299), but had little effect on the ratio of SD between the adaxial and abaxial surfaces (*P* = 0.803, *F* = 0.033, df = 299). The adaxial/abaxial ratio of SD was almost a constant, with a value of 0.73 in ambient temperature and 0.72 in elevated temperature (Table 3). However, we found that warming significantly increased the adaxial/abaxial ratio of SD and SI by 18.1% and 30.6% at the base section (all *P* < 0.05), while had little effect on those at the tip and middle sections of the maize leaves (all *P* > 0.05; Table 3).

**Table 3:** Effects of warming on the ratio of stomatal density (SD), epidermal cell density (ECD), and stomatal density (SI) between adaxial and abaxial surfaces of maize leaves

	Ambient temperature			Elevated temperature				
				
Adaxial/abaxial ratio	Tip	Middle	Base	Tip	Middle	Base	Increase (%)	*P*-value
SD	0.69 (0.07)^bc^	0.77 (0.10)^ab^	0.72 (0.09)^b^	0.61 (0.06)^c^	0.70 (0.08)^bc^	0.85 (0.12)^a^	–	–
	0.73 (0.09)			0.72 (0.13)			−1.4	*P =* 0.803
ECD	1.07 (0.06)^ab^	1.16 (0.12)^a^	1.06 (0.05)^ab^	0.99 (0.13)^bc^	0.93 (0.10)^c^	0.92 (0.04)^c^	–	–
	1.10 (0.09)			0.95 (0.10)			−13.6	*P* < 0.001
SI	0.70 (0.07)^bc^	0.72 (0.13)^bc^	0.72 (0.09)^bc^	0.67 (0.10)^c^	0.80 (0.09)^b^	0.94 (0.12)^a^	–	–
	0.71 (0.09)			0.80 (0.15)			12.3	*P* = 0.028

Values given are means ± standard deviation for five subsamples and three replicates. Mean values were compared by the one-way analysis of variance (ANOVA) at *P* < 0.05. Different letters indicate *P* < 0.05 and the same letters indicate *P* > 0.05.

### Stomatal aperture size and shape

**Figure 1:**
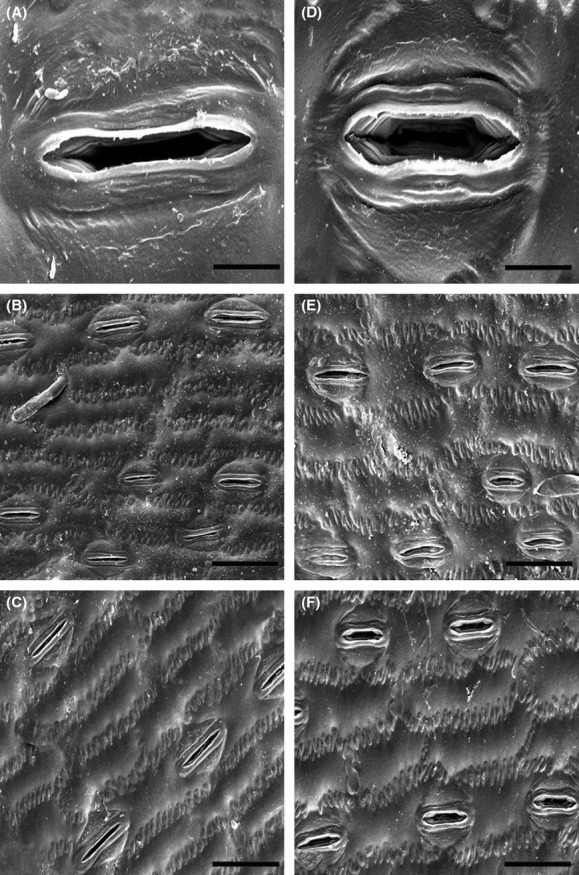
Scanning electron micrographs (SEM) showed the characteristics of stomata and epidermal cells at the middle section of maize leaves grown in ambient (A–C) and elevated temperature (D–F). Note that shorter and wider stomatal pores were observed on both the adaxial surface (B) and abaxial surface (C) of maize leaves grown at elevated temperature than those of their counterparts (E and F) grown at ambient temperature. In addition, elevated temperature also increased the width of epidermal cells. Bars, 10 μm (A and D) and 40 μm (B, C, E, and F).

Experimental warming not only affected stomatal frequency but also changed individual stomatal size. Our results showed that warming significantly decreased stomatal aperture length, but increased stomatal aperture width (Table 1). We found that warming decreased stomatal aperture length from 35.9 μm to 29.8 μm, about 17.0% (*P* < 0.001, *F* = 7.010, df = 149; Table 1). Specifically, warming significantly decreased stomatal aperture length at the tip, middle, and base sections by 15.7% (*P* = 0.032, *F* = 6.158, df = 149), 16.0% (*P* < 0.001, *F* = 135.652, df = 149), and 9.7% (*P* = 0.005, *F* = 13.228, df = 149) on the adaxial surface and by 21.4% (*P* < 0.001, *F* = 2.767, df = 149), 14.9% (*P <* 0.001, *F* = 78.053, df = 149), and 23.0% (*P* < 0.001, *F* = 121.950, df = 149) on the abaxial surface (Table 2). By contrast, experimental warming significantly increased the stomatal aperture width from 3.4 μm to 4.6 μm, about 33.8% (*P <* 0.001, *F* = 31.660, df = 449; Table 1). Meanwhile, experimental warming also had different effects on stomatal aperture width among leaf sections (Table 2). Compared with the ambient temperature, the stomatal aperture width was significantly increased 32.4% (*P* = 0.010, *F* = 10.114, df = 149) at the middle section of the adaxial surface and 48.4% (*P* = 0.013, *F* = 9.152, df = 149) and 51.4% (*P* < 0.001, *F* = 4.910, df = 149) at the middle and base sections of the abaxial surface under elevated temperature (Table 2). These results were also confirmed by our directly scanning electron microscopic observation where we compared the microscopic images of leaves grown under elevated and ambient temperatures. We also observed shorter and wider stomata on both adaxial and abaxial surfaces of leaves grown at elevated temperature than those leaves grown at ambient temperature (Fig. 1).

As a result, warming significantly increased the stomatal aperture area (SAA) by 30.1% (*P* < 0.001, *F* = 38.531, df = 449) and stomatal aperture area index (SAAI), defined as the stomatal aperture area per unit leaf area, by 39.9% (*P* < 0.001, *F* = 35.940, df = 449; Table 1). We found that warming significantly increased the SAA and the SAAI at various leaf sections on both leaf surfaces (all *P* < 0.05) except for the tip section on the adaxial surface (Table 2). Meanwhile, warming also significantly increased stomatal aperture circumference (SAC) by 13.1% (*P =* 0.003, *F* = 9.777, df = 449; Table 1). However, we found that warming had different effects on the SAC between leaf surfaces and among leaf sections (Table 2). Specifically, warming significantly increased the SAC by 28.4% (*P* = 0.005, *F* = 10.523, df = 149) at the tip leaf section but had little effect on the middle and base sections on the adaxial surfaces. In contrast to the adaxial surface, warming significantly increased the SAC by 20% (*P* < 0.001, *F* = 25.860, df = 149) and 10.2% (*P* = 0.032, *F* = 16.352, df = 149) at the middle and base sections on the adaxial surface, whereas only little warming effect was detected at the tip section (Table 2). Moreover, our results also showed that warming had little effect on the SASI (Table 1). We found that warming barely affected the SASI at various leaf sections including tip, middle, and base section on both leaf surfaces except for the tip section on the abaxial leaf surface. However, warming significantly increased the SASI by 16.3% (*P* = 0.021, *F* = 15.263, df = 149) at the tip section on the abaxial surface of maize leaves (Table 2).

Our three-way ANOVA results showed that temperature, leaf surface, and leaf section had significantly interactive effects on stomatal features of maize leaves (Table 4). We found that temperature × leaf section had significantly effects on the SI (*P* = 0.002, *F* = 5.229, df = 449), SAL (*P* < 0.001, *F* = 24.391, df = 449), and SAAI (*P* = 0.008, *F* = 4.680, df = 449), while temperature × leaf surface only affected the SI (*P* = 0.042, *F* = 8.136, df = 449) and SAAI (*P* = 0.006, *F* = 6.998, df = 449). Leaf surface × leaf section significantly affected the SD (*P* = 0.019, *F* = 3.727, df = 449), SI (*P* = 0.013, *F* = 3.910, df = 449), and SAW (*P* = 0.018, *F* = 6.083, df = 449). Meanwhile, we also found that temperature × leaf surface × leaf section significantly changed the SD (*P* = 0.025, *F* = 4.432, df = 449), SAC (*P* = 0.002, *F* = 5.019, df = 449), SAAI (*P* = 0.01, *F* = 8.420, df = 449), and SASI (*P* = 0.006, *F* = 4.865, df = 449) of maize leaves.

**Table 4:** Warming effects on stomatal characteristics at different leaf surfaces and sections of maize plants

Parameters	SD	SI	SAL	SAW	SAA	SAC	SAAI	SASI
Temperature	**0.038**	**<0.001**	**<0.001**	**<0.001**	**<0.001**	**<0.001**	**<0.001**	0.756
Leaf surface	**<0.001**	**<0.001**	0.061	0.982	**<0.001**	**<0.001**	**<0.001**	0.861
Leaf section	**<0.001**	**<0.001**	**<0.001**	**<0.001**	**<0.001**	0.583	**<0.001**	**<0.001**
Temperature × leaf surface	0.608	**0.042**	0.628	0.873	0.409	0.512	**0.006**	0.281
Temperature × leaf section	0.307	**0.002**	**<0.001**	0.357	0.076	0.658	**0.008**	0.811
Leaf surface × leaf section	**0.019**	**0.013**	0.359	**0.018**	0.421	0.253	0.428	0.225
Temperature × leaf surface × leaf section	**0.025**	0.083	0.722	0.774	0.090	**0.002**	**0.010**	**0.006**

Values given are means ± standard deviation for SD, SI (25 subsamples and three replicates) and for SAL, SAW, and SAA (150 subsamples and three replicates). Mean values were compared by the ANOVA followed by Duncan's multiple range test, and the different letters represent statistical differences at *P* < 0.05. The bold value indicates *P* < 0.05. SD: Stomatal density (number per mm^2^); ECD: Epidermal cell density (number per mm^2^); SI: Stomatal index (%); SAL: Stomatal aperture length (μm); SAW: Stomatal aperture width (μm); SAA: Stomatal aperture area (μm^2^); SAAI: Stomatal aperture area index (%) which is the total stomatal aperture area per unit leaf area calculating as stomatal average density × stomatal aperture area per stoma.

### Spatial distribution pattern of stomata

**Figure 2:**
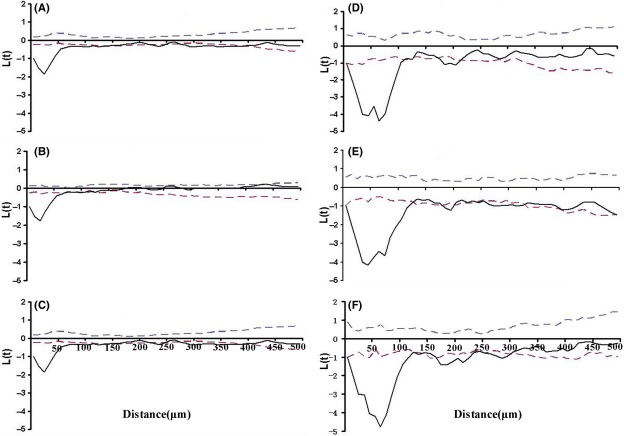
Point pattern analyses of stomata on the adaxial surface in leaf 1, leaf 2, and leaf 3 of maize plants grown at ambient temperature (A–C) and in leaf 1, leaf 2, and leaf 3 of maize plants grown at elevated temperature (D–F), respectively. The dotted lines give a 95% confidence envelope for complete spatial randomness. The data were given for three leaves from three ambient or warmed plots.

**Figure 3:**
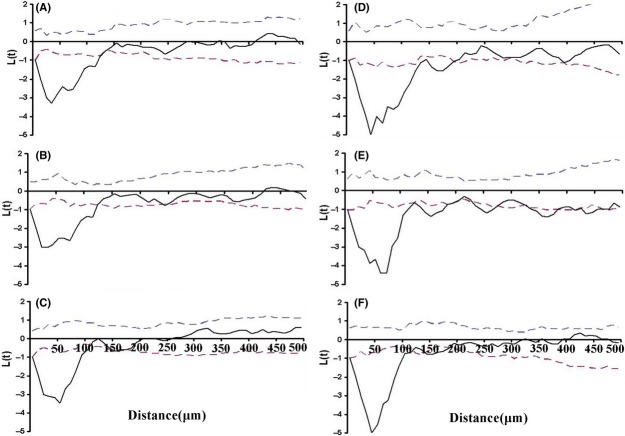
Point pattern analyses of stomata on the abaxial surface in leaf 1, leaf 2, and leaf 3 of maize plants grown at ambient temperature (A–C) and in leaf 1, leaf 2, and leaf 3 of maize plants grown at elevated temperature (D–F), respectively. The dotted lines give a 95% confidence envelope for complete spatial randomness. The data were given for three leaves from three ambient or warmed plots.

We found that the stomata on maize leaves followed a regular distribution at small scales (<140 μm) and a random distribution at larger scales for both adaxial and abaxial leaf surfaces and for leaves grown in both ambient and warming plots (Fig. 2). However, the stomata on the abaxial surfaces tended to be more regular than those on the adaxial surfaces because the abaxial surface had lower *L*(*t*) values than the adaxial surface at the same scale, especially for the leaves grown in the ambient temperature (*P* < 0.001, *F* = 109.5, df = 5; Figs 2 and 3). The most regular pattern occurred at a spatial scale of *c*. 25 μm with the average minimum *L*(*t*) value of −1.8 for the adaxial surface and −3.3 for the abaxial surface for leaves grown in the ambient temperature (Fig. 2A–C). We also found that warming made the stomata more regularly distributed on both leaf surfaces because the average minimum *L*(*t*) value of the warmed leaves was significantly lower than that of the leaves in the ambient temperature. Specifically, warming significantly decreased the average minimum *L*(*t*) value from −1.81 to −4.43 for the adaxial surface (*P* < 0.001, *F* = 241.9, df = 5; Fig. 2) and from −3.25 to −4.80 for the abaxial surface (*P* = 0.003, *F* = 41.399, df = 5; Fig. 3). Moreover, warming also increased the scale range of the regular distribution with the most regular pattern occurred at a scale of *c*. 60 μm for the warmed leaves and only *c*. 30 μm for the control leaves (Figs 2 and 3). This warming effect on stomatal distribution pattern, in general, was greater on the adaxial surface than the abaxial surface and the warming effect was consistent among all the replicate leaves (Figs 2 and 3).

### Stomatal conductance and transpiration rate

**Figure 4:**
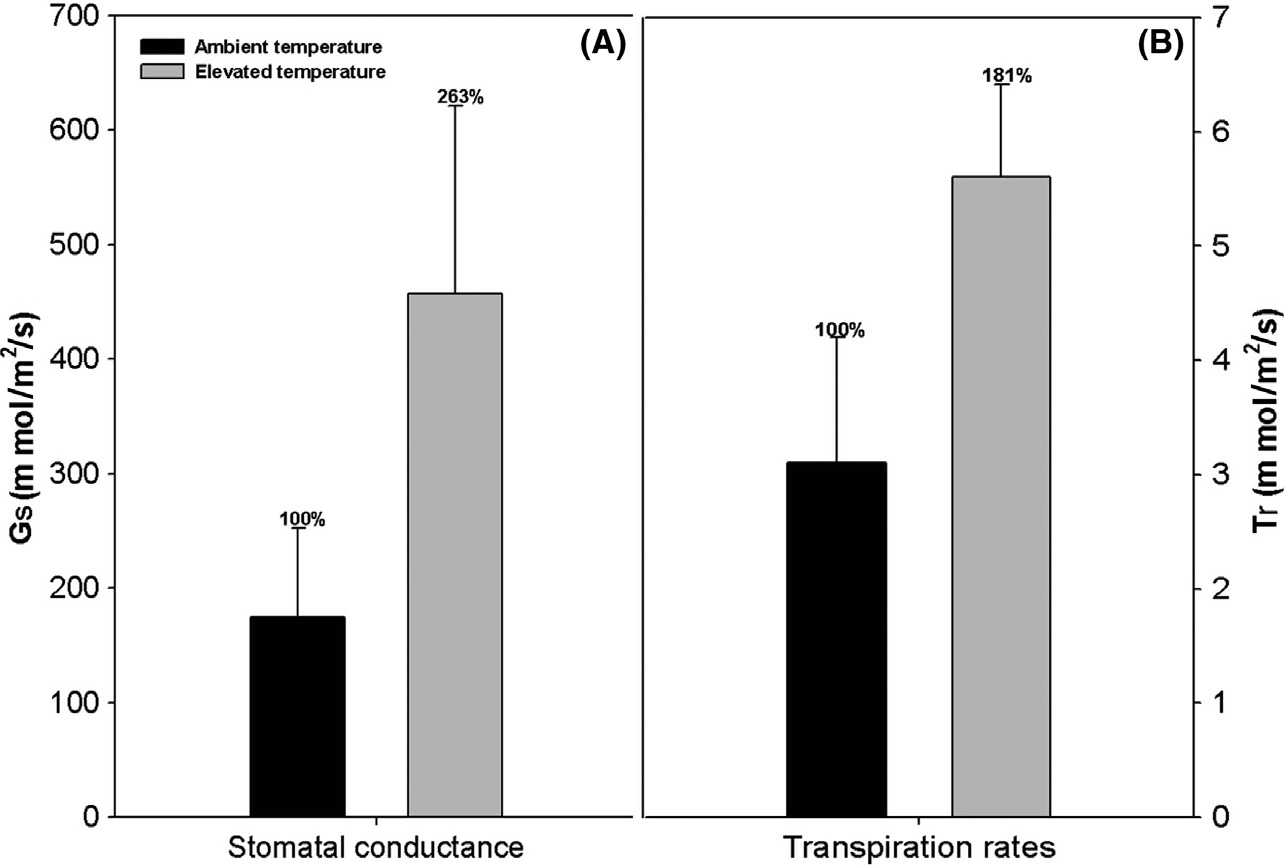
Stomatal conductance (A) and transpiration rate (B) of maize leaves grown under ambient temperature or elevated temperature. The data given are means ± standard deviation for five leaves from three ambient or warmed plots. Note that experimental warming significantly increased stomatal conductance (Gs) and transpiration rate (Tr) 163% and 81%, respectively.

Experimental warming not only changed stomatal traits but also increased stomatal conductance (Gs) and transpiration rate (Tr) of maize leaves (Fig. 4). We found that warming significantly increased stomatal conductance from 174 to 457 mmol/m^2^/s by 163% (*P* = 0.001, *F* = 16.970; df = 9; Fig. 4). Similarly, transpiration rate was also increased by 181% (from 3.1 to 5.6 mmol/m^2^/s) under warming conditions (*P* = 0.017, *F* = 8.483; df = 9; Fig. 4).

## Discussion

### Warming effects on stomatal density and stomatal index

The responses of stomatal density (SD, the number of stomata per unit area) and stomatal index (SI, the proportion of stomata in relation to total number of epidermal plus stomatal cells) to global warming are important in determining the potential efficiency of leaf-level gas exchange, and thus ecosystem carbon cycles under future global warming (Woodward 1987; Ferris et al. 1996; Apple et al. 2000). In this study, we found that warming had little effect on SD but significantly increased SI of the leaves of maize plants (*Zea may* L.) because warming significantly decreased the number of epidermal cells and meanwhile had no effect on the number of guard cells. Previous studies have reported that increasing temperature enhanced epidermal cell expansion on maize leaves but had little effect on the epidermal cell division (Erwin et al. 1991, 1994; Tardieu et al. 2000). This suggests that the decrease of the number of epidermal cells per unit leaf area in the warming plots in the current study was mainly due to the greater expansion of the individual epidermal cells, rather than greater differentiation to guard cells under the warming treatment. Our results also confirmed that the average size of the individual epidermal cells was significantly larger in the warming plots than in the control plots (Fig. 1 and Table S1), resulting in the asymmetric warming effect on guard cells and epidermal cells on the maize leaves. Similarly, several studies have also found that plant leaves respond to elevated CO_2_ concentration with adjusting their stomatal densities through changes in epidermal cell numbers rather than stomatal numbers (Ferris et al. 2002; Driscoll et al. 2006; Soares et al. 2008).

In addition to temperature, stomatal frequency is also affected by other environmental factors, such as drought, irradiance, light intensity, and relative air humidity (Apple et al. 2000; Fraser et al. 2009; Xu et al. 2009). The inconsistent conclusions in the literatures concerning warming effects on stomatal frequency may result from different warming methods, different warming intensity and possibly different species. Different warming methods, such as using open-top chamber (Fraser et al. 2009), greenhouse (Ferris et al. 1996; Reddy et al. 1998; Apple et al. 2000; Luomala et al. 2005; Xu and Zhou 2005), and growth chamber (Hovenden 2001; Xu et al. 2009; Jin et al. 2011), may alter other environmental factors, such as soil water content, light intensity, and relative humidity, in addition to temperature. For example, Niu et al. (2007) compared the disadvantages of different warming facilities for simulating climate warming and concluded that open-top chamber and greenhouse altered the microclimates including light, humidity, and rainfall inside them except for temperature. Luomala et al. (2005) pointed out that higher VPD inside the elevated temperature growth chambers during growth season might affect stomatal density. Moreover, several studies have compared stomatal density of plant leaves growing in different geographical locations (Beerling and Chaloner 1993), and altitudinal gradients (Kouwenberg et al. 2007) with different air temperatures for simulating climate warming, which apparently changed climate conditions such as precipitation, soil moisture, and light intensity and period. As a result, the different growth conditions may contribute to the inconsistent results of warming effects among species and ecosystems. The current study examined the warming effects on the stomatal frequency of maize leaves with an infrared radiator in field conditions, which had little disturbance on the microclimates and few difference in relative air humidity between the control and warmed plots (Niu et al. 2007; Zhang et al. 2010).

### Warming effects on stomatal aperture size and shape

Plants in response to climate warming not only alter stomatal frequency (Apple et al. 2000; Luomala et al. 2005; Xu and Zhou 2005), but also change stomatal aperture size anatomically (Hetherington and Woodward 2003; Franks and Beerling 2009; Casson and Hetherington 2010). It is noted that the stomatal aperture size here refers to the stomatal aperture length which is the linear distance between the junctions of the guard cells at each end of the stomata. The stomatal aperture size is different from stomatal pore openness which responds simultaneously to environmental variables such as light (Humble and Hsiao 1970; Sharkey and Raschke 1981; Kwak et al. 2001; Takemiya et al. 2006), temperature (Honour et al. 1995; Feller 2006; Reynolds-Henne et al. 2010), humidity (Lange et al. 1971; Schulze et al. 1974), and CO_2_ concentration (Ogawa 1979; Young et al. 2006; Lammertsma et al. 2011). Anatomically, the length of guard cells mainly determines stomatal aperture length, because when stomata open or close the short axis (ventral and dorsal lengths) of the guard cells can increase or decrease but the long axis remains the same (Willmer and Fricker 1996; Beaulieu et al. 2008). Several studies have found that stomatal aperture size was regulated and modified by environment factors such as CO_2_ concentration (Hetherington and Woodward 2003; Franks and Beerling 2009; Casson and Hetherington 2010). However, recent studies found a strong positive relationship between angiosperm genome size (nuclear DNA amount) and stomatal guard cell length and this predictive relationship was independent of environmental conditions (Beaulieu et al. 2008; Lomax et al. 2009). For example, Lomax et al. (2009) examined the effects of environmental variables (CO_2_ concentration, drought, relative humidity, irradiance, ultraviolet radiation, and pathogen attack) on the guard cell length of *Arabidopsis thaliana* and found that guard cell length responded to all these variables, but the predictive relationship between genome size and guard cell length was not changed by these environmental variables. Unfortunately, the study did not examine the temperature effect on the guard cell length. Interestingly, in the current study, we found that warming significantly reduced the guard cell length (Table S1), thus resulting in the reduction in stomatal aperture length (Table 1 and Fig. 1). This finding supports a recent study that experimental warming significantly decreased the stomatal aperture length of four alpine grass species (Zhang et al. 2010). It is noted that the decrease in stomatal length with warming is accompanied with the increase in the optimal stomatal aperture width (measured at optimal conditions). As a result, we found that warming significantly increased the stomatal aperture area (SAA) and the SAA index (*P* < 0.01; Table 1). This finding is also supported by our measurements of leaf stomatal conductance which was significantly higher in the warming plots than the control plots (Fig. 4).

### Warming effects on spatial distribution pattern of stomata

Previous studies have suggested that the dorsoventral (adaxial/abaxial) polarity of maize leaf is established in the meristem and is subsequently maintained throughout leaf development stages (Juarez et al. 2004; Driscoll et al. 2006). Our results showed that warming had little effect on the adaxial/abaxial ratio in SD (*P* = 0.856; Table 3), suggesting that the adaxial/abaxial polarity of the SD in maize leaves is genetically controlled and independent of the changes in environmental condition such as warming. Similar results have been reported in maize plants under CO_2_ enrichment conditions by Driscoll et al. (2006), who found that the dorsoventral pattern (adaxial/abaxial) of stomata in maize plants is independent of and not affected by CO_2_ concentrations. Moreover, we also found that the adaxial/abaxial ratio of SI was significantly increased by experimental warming (*P* = 0.037; Table 3), which was mainly due to the decrease of the ECD ratio between the adaxial and abaxial surfaces (*P* < 0.001; Table 3). These results suggest that maize plants in response to global warming may alter the dorsoventral distribution of stomata by changing the adaxial/abaxial ratio of ECD.

In addition to the stomatal frequency between the adaxial and abaxial leaf surfaces, experimental warming also resulted in uneven effects on stomatal features along different sections such as tip, middle, and base within a leaf surface (Table 2). These results suggested that the warming effects feature high within-surface and within-leaf variations in stomatal dimensions, distribution, and characteristics of maize leaves. Similar results were also found in two perennial grasses, *Lolium perenne* (Ferris et al. 1996) and *Leymus chinensis* (Xu et al. 2009). However, many previous studies examined warming effects on stomatal features only at the middle section on the abaxial leaf surface (Beerling and Chaloner 1993; Hovenden 2001; Xu and Zhou 2005; Kouwenberg et al. 2007). Therefore, it is noted that the sampling method of stomata should be developed for evaluating the stomatal characters across the whole leaf.

Experimental warming not only changes stomatal distribution features at a leaf scale but also affects spatial distribution pattern of stomata at a smaller scale in leaf sections. Previous studies mainly focused on the one-dimensional pattern of stomatal distribution (Juarez et al. 2004; Shpak et al. 2005; Wang et al. 2007) or the behavior of a single stoma (Shimazaki et al. 2007; Shang et al. 2009), because it is difficult to characterize the spatial distribution pattern of stomata (Croxdale 2000; Martins et al. 2012). In this study we examined the warming effects on the spatial distribution pattern of stomata using the geostatistical method (Ripley's *K*-function), which is considered as an accurately mathematical technique for analyzing spatial distribution patterns (Skarpe 1991; Haase et al. 1997). We observed a more regular spatial distribution pattern of stomata in elevated temperature than that in ambient temperature. This suggested that experimental warming may enhance leaf gas exchange efficiency of maize plants, because the most regular distribution pattern of stomata features the shortest CO_2_ diffusion distance to other stomata. In the current study, we also found that warming increased stomatal conductance and transpiration rate (Fig. 4) which was partly attributed to the more regular spatial distribution pattern of the stomata. This warming effect on stomatal spatial distribution pattern may also be a strategy for plants to adapt to global warming because the increased transpiration with the increase of stomatal conductance can cool the leaves, especially in the hot summer with daytime temperature around 40°C in northern China.

### Warming, stomata, and C_4_ photosynthetic pathway evolution

Many of the most productive crops such as maize and sugarcane use the C_4_ photosynthetic pathway, which offers C_4_ plants the potential to achieve higher rates of leaf photosynthesis and more efficient use of water and nitrogen than C_3_ plants (Osborne and Freckleton 2009; Taylor et al. 2012). In comparison with C_3_ plants, the higher photosynthetic capacity of C_4_ plants is mainly due to their unique mode of CO_2_ assimilation, featuring strict compartmentation of photosynthetic enzymes into two distinct cell types, mesophyll and bundle sheath (Wang et al. 2009). In this study, our results showed that experimental warming not only increased individual size of stoma, but also resulted in more regular spatial distribution pattern of stomata in maize leaves, which may reduce the CO_2_ diffusion distance from each stoma to photosynthetic site and thus increased the photosynthesis rates of maize plants. These results suggested that global warming may improve the evolution of C_4_ photosynthesis through the changes in stomatal traits including stomatal frequency, stomatal size, and spatial distribution pattern of stomata. Moreover, our findings also help to better understand the role of stomatal changes in the long-term evolution of wild C_4_ crop progenitors in a subambient CO_2_ condition from the origin of agriculture (Sage 1995; Cunniff et al. 2008; Aliscioni et al. 2012). In addition, the enhancement in C_4_ photosynthesis efficiency may increase the aboveground biomass accumulation of C_4_ plants (Luo et al. 2009; Hou et al. 2012). For example, Luo et al. (2009) showed that experimental warming stimulates aboveground biomass accumulation through enhancing C_4_ dominance in a North America tallgrass prairie. Therefore, our results suggested that future global warming may affect the contribution of agroecosystems to CO_2_ sequestration in a warmer world.

## Supporting Information

**Table S1.** The characteristics of epidermal cells and guard cells in maize leaves grown at ambient temperature or elevated temperature.
