# Supplementary material for: Effects of experimental warming on stomatal traits in leaves of maize (Zea may L.)
Source: Ecol Evol. 2013 Aug 1;3(9):3095–111. doi: 10.1002/ece3.674 (PMC3790554; doi:10.1002/ece3.674)
Supplement: Supplementary file 1 [file ece30003-3095-SD1.docx]

**Suplimental information 1. The characteristics of epidermal cells and guard cells in maize leaves grown at ambient temperature or elevated temperature.**

| **Parameters** | Ambient temperature | | Elevated temperature | | | Increase  (%) | *P*-value |
| --- | --- | --- | --- | --- | --- | --- | --- |
|  | Adaxial | Abaxial | Adaxial | Abaxial | |  |  |
| **Epidermal cell (EC) features** | | | | | | | |
| ECD (number/mm^2^) | 349(27)^a^ | 321(11)^ab^ | 296(28)^b^ | 314(16)^ab^ | | — | — |
|  | 335 | | 305 | | | -9.0 | *P*<0.05 |
| ECL (µm) | 90.7(5.4)^a^ | 74.5(14.3)^b^ | 94.3(19.0)^a^ | 82.6(16.4)^ab^ | | — | — |
|  | 81.7 | | 89.3 | | | 9.3 | *P*>0.05 |
| ECW (µm) | 24.0(3.0)^bc^ | 21.9(4.4)^c^ | 29.1(4.4)^a^ | 25.6(3.0)^b^ | | — | — |
|  | 22.9 | | 27.6 | | | 20.6 | *P*<0.01 |
| ECA (μm^2^) | 2104(326)^b^ | 1496(309)^c^ | 2638(551)^a^ | 2051(392)^b^ | | — | — |
|  | 1766 | | 2384 | | | 35.0 | *P*<0.001 |
| ECC (µm) | 247(24)^a^ | 183(26)^b^ | 293(82)^a^ | 249(54)^a^ | | — | — |
|  | 212 | | 274 | | | 29.6 | *P*<0.001 |
| **Guard cell (GC) features** | | | | | | | |
| GCL (μm)** | 52.4(4.3)^a^ | 49.8(4.0)^b^ | 47.1(3.3)^c^ | | 42.2(2.6)^d^ | — | — |
|  | 51 | | 45 | | | -11.8 | *P*<0.05 |
| GCW (μm)** | 6.2(2.87)^d^ | 8.3(1.24)^c^ | 11.6(1.6)^b^ | | 12.6(1.2)^a^ | — | — |
|  | 7.3 | | 12.1 | | | 66 | *P*<0.001 |
| GCA (μm^2^) | 453(78)^b^ | 468(77)^ab^ | 497(70)^ab^ | | 504(72)^a^ | — | — |
|  | 461 | | 501 | | | 8.0 | *P*>0.05 |
| GCC (μm) | 126(11)^a^ | 129(9)^a^ | 130(15)^a^ | | 134(22)^a^ | — | — |
|  | 128 | | 132 | | | 3.1 | *P*>0.05 |

Values given are means ± standard deviation for ECD (75 subsamples, 3 replicates) and for other parameters (450 subsamples, 3 replicates). Mean values were compared by the ANOVA followed by Duncan’s multiple range test, and the different letters represent statistical differences at *P*<0.05. ECD: Epidermal cell density; ECL: Epidermal cell length; ECW: Epidermal cell width; ECA: Epidermal cell area; ECC: Epidermal cell circumference; GCL: Guard cell length; GCW: Guard cell width; GCA: Guard cell area; GCC: Guard cell circumference. **Guard cell length is the longest dimension, and the guard cell width is the widest dimension.
